# Supplementary material for: Prefusion-stabilized SARS-CoV-2 S2-only antigen provides protection against SARS-CoV-2 challenge
Source: Nat Commun. 2024 Feb 20;15:1553. doi: 10.1038/s41467-024-45404-x (PMC10879192; doi:10.1038/s41467-024-45404-x)
Supplement: Supplementary file 1 — Supplementary Information [file 41467_2024_45404_MOESM1_ESM.pdf]

Supplementary Information for

Prefusion-stabilized SARS-CoV-2 S2-only antigen provides protection against SARS-CoV-2 challenge

Ching-Lin Hsieh[1#], Sarah R. Leist[2#], Emily Happy Miller[3], Ling Zhou[1], John M. Powers[2], Alexandra L. Tse[3], Albert Wang[3], Ande West[2], Mark R. Zweigart[2], Jonathan C. Schisler[4], Kartik Chandran[5], Ralph S. Baric[2], Jason S. McLellan[1]

[1] Department of Molecular Biosciences, The University of Texas at Austin, Austin, TX, USA 78712

[2] Department of Epidemiology, University of North Carolina at Chapel Hill, Chapel Hill, NC, USA 27599

[3] Department of Microbiology and Immunology, Albert Einstein College of Medicine, Bronx, NY, USA 10461

[4] McAllister Heart Institute and Department of Pharmacology, The University of North Carolina at Chapel Hill, USA 27599

[5] Department of Medicine-Infectious Diseases, Albert Einstein College of Medicine, Bronx, NY, USA 10461

[#]Authors contributed equally

*Correspondence: jmclellan@austin.utexas.edu (J.S.M.)

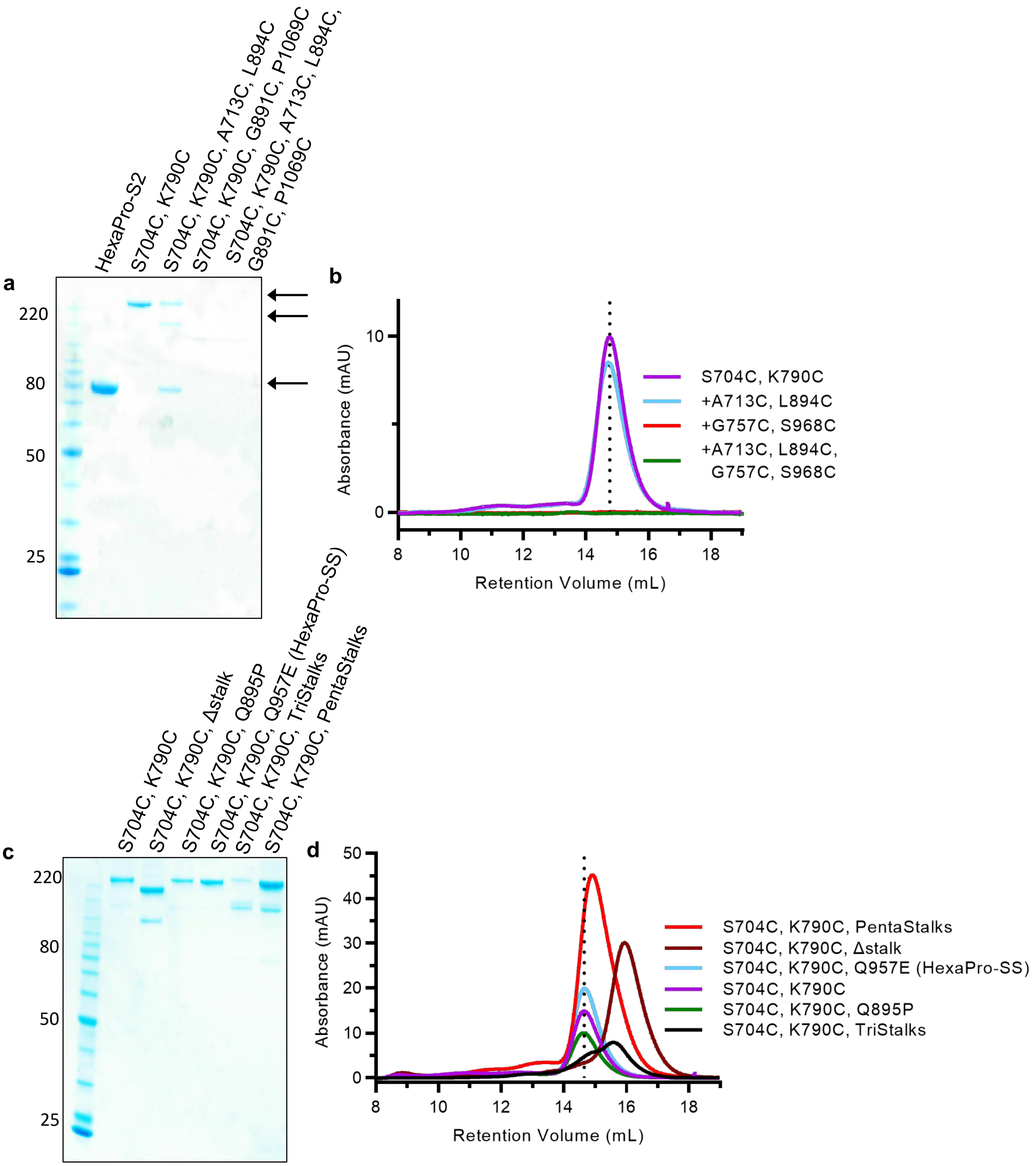

Supplementary Fig. 1. Characterization of small-scale expression of S2-only variants. **a,** Non-reducing SDS-PAGE analysis of each interprotomer disulfide variants. **b,** SEC profile of affinity-purified interprotomer disulfide variants from 40 ml cultures that were applied to to Superose 6 increase 10/300. The molecular weight standards in kDa are indicated at the left. The position of monomer, dimer and trimer bands are indicated at the right. **c,** Non-reducing SDS-PAGE analysis of each S2 combinatorial variants. **d,** SEC profile of affinity-purified S2 combinatorial variants from 40 ml cultures that were applied to to Superose 6 increase 10/300. The vertical dotted line indicates the peak retention volume (**b,d**) for S704C, K790C variant. The SDS-PAGE analysis ran one time.

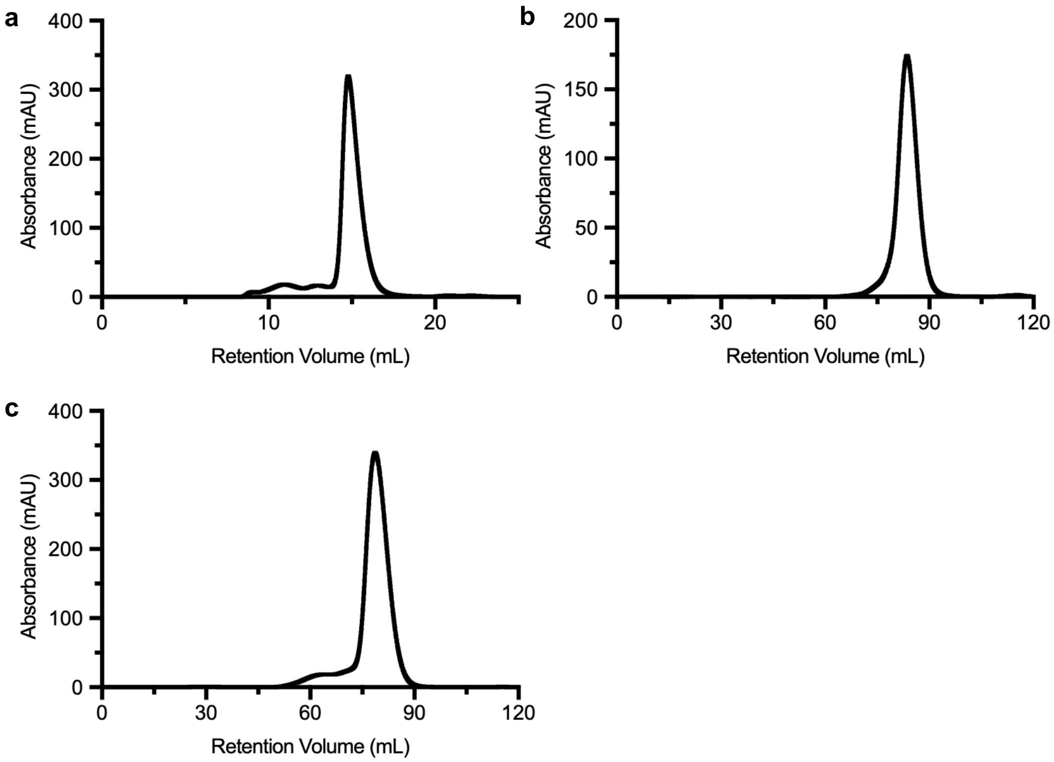

Supplementary Fig. 2. SEC profile of large-scale expression of HexaPro-SS variants. a, Affinity-purified HexaPro-SS from 1-L culture was applied to to Superose 6 increase 10/300. **b-c,** Affinity-purified (**b**) HexaPro-SS-Δstalk from 0.5-L culture and (**c**) HexaPro-SS-PentaStalks were applied to Superose 6 16/70, respectively.

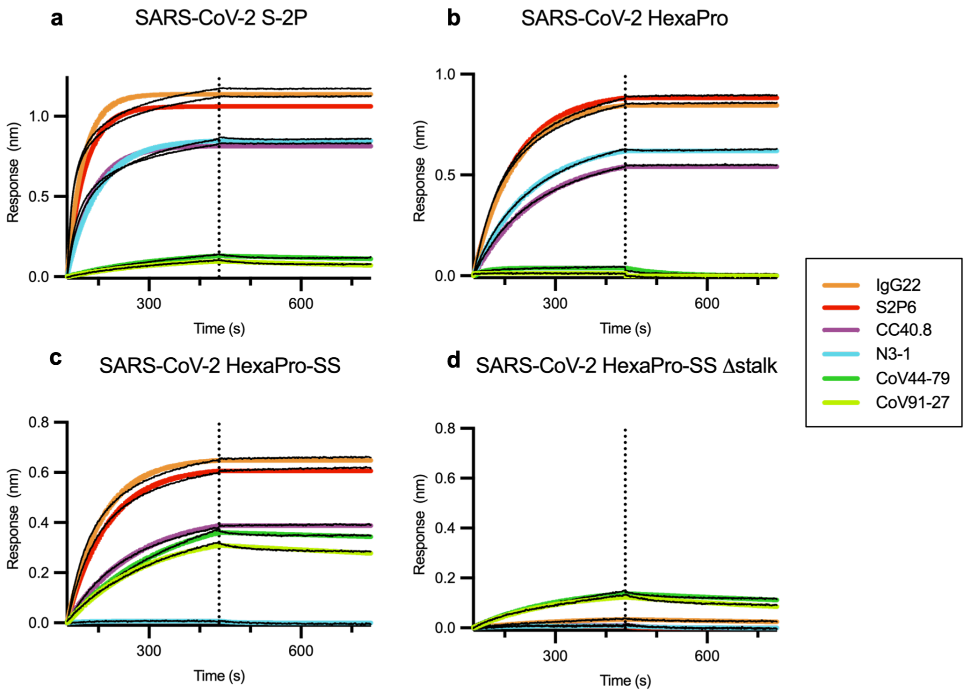

Supplementary Fig. 3. Biolayer interferometry binding characterization confirms epitope preservation on HexaPro-SS and HexaPro-SS Δstalk. A panel of S2-directed antibody IgGs (stem helix binders: IgG22, S2P6, and CC40.8; fusion peptide binders: CoV44-79 and CoV91-27) were used to test binding to SARS-CoV-2 S-2P (**a**), SARS-CoV-2 HexaPro (**b**), SARS-CoV-2 HexaPro-SS (**c**), and SARS-CoV-2 HexaPro-SS Δstalk (**d**). Antibody IgG N3-1 is an RBD-directed antibody that acts as an S1-binding control in the experiment.

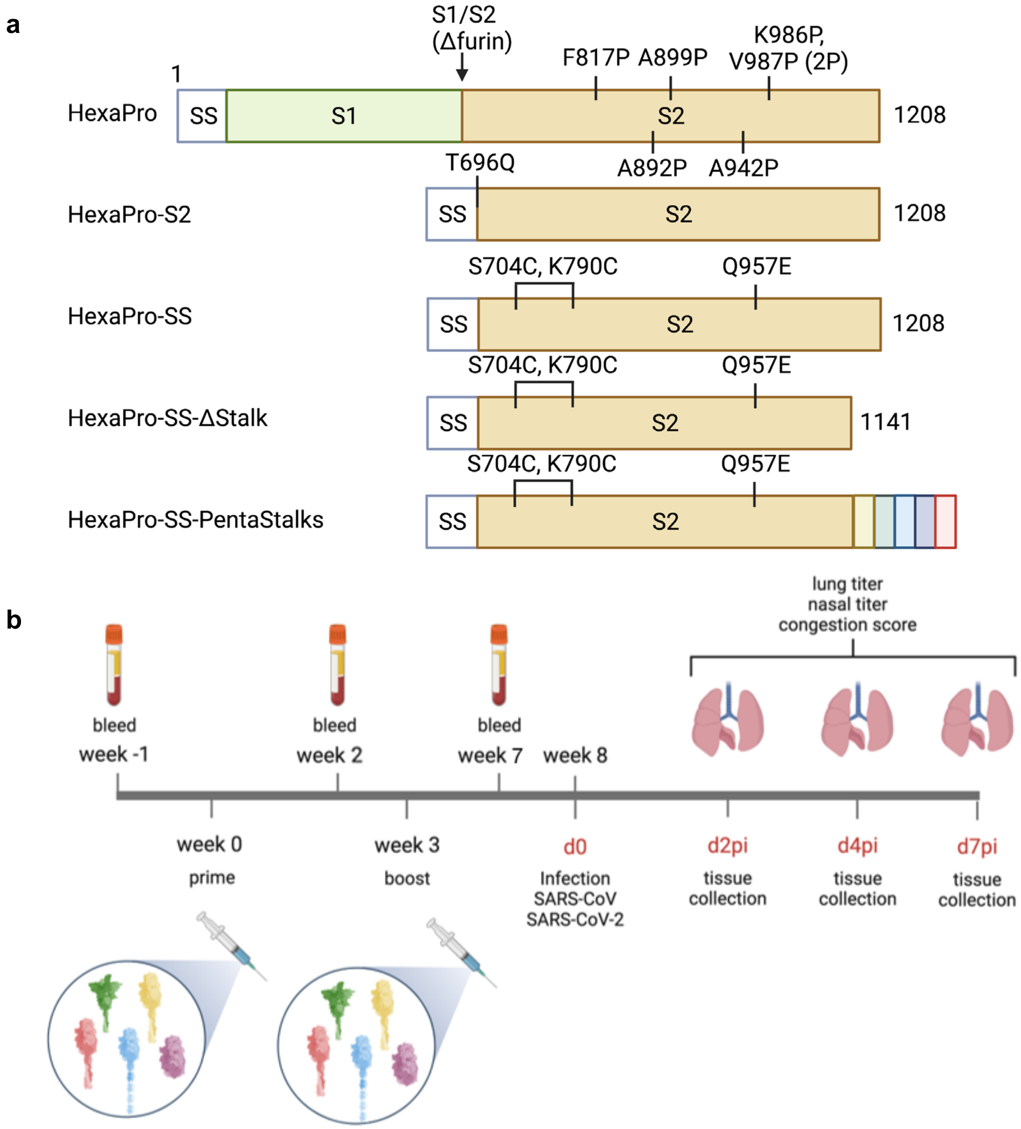

Supplementary Fig. 4. Schemes of immunogenicity study. a, The primary sequence of the immunogens with substitutions highlighted. **b,** BALB/cAnNHsd mice were primed and boosted with 10 μg of respective S immunogens, and the blood was collected for assessing serum neutralization titers. Mouse body weight was monitored until day 7 after infection (d7pi), nasal and lung viral titers and congestion scores were measured at indicated time points (d2pi, d4pi, d7pi). The cartoons were created by BioRender.com.

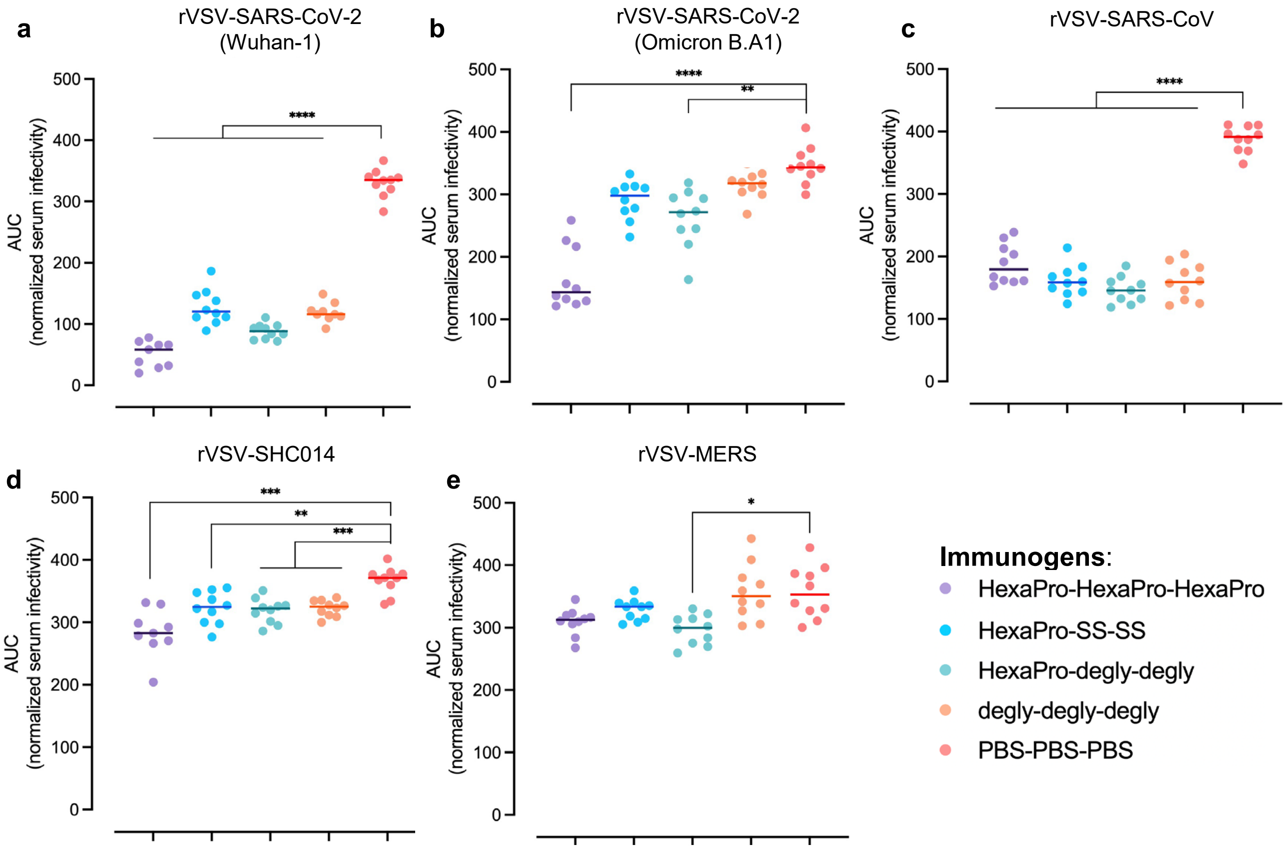

Supplementary Fig. 5. Sera from mice double boosted with S2 immunogens broadly neutralize a range of rVSV-CoVs a-e, Pre-titrated amounts of rVSV-CoVs were incubated with serial 3-fold dilutions of sera from mice (n=10/immunized group) immunized with respective S antigens or PBS at RT for 1 hour. Virus-sera mixtures were then added to monolayers of Vero cells. At 10hr post-infection, cells were fixed, and nuclei were counterstained. Infected cells were scored using BioTek Cytation5 for presence of GFP. Sera from 10 mice are included in each group. Area under the curve (AUC) was calculated from normalized infectivity levels. One-way ANOVA (**a,c**), Welch's ANOVA (**d,e**) or Kruskal-Wallis test (**b**) were run based on normality and homoscedasticity. **** $p<0.0001$, *** $p < 0.001$, ** $p < 0.01$, * $p < 0.05$

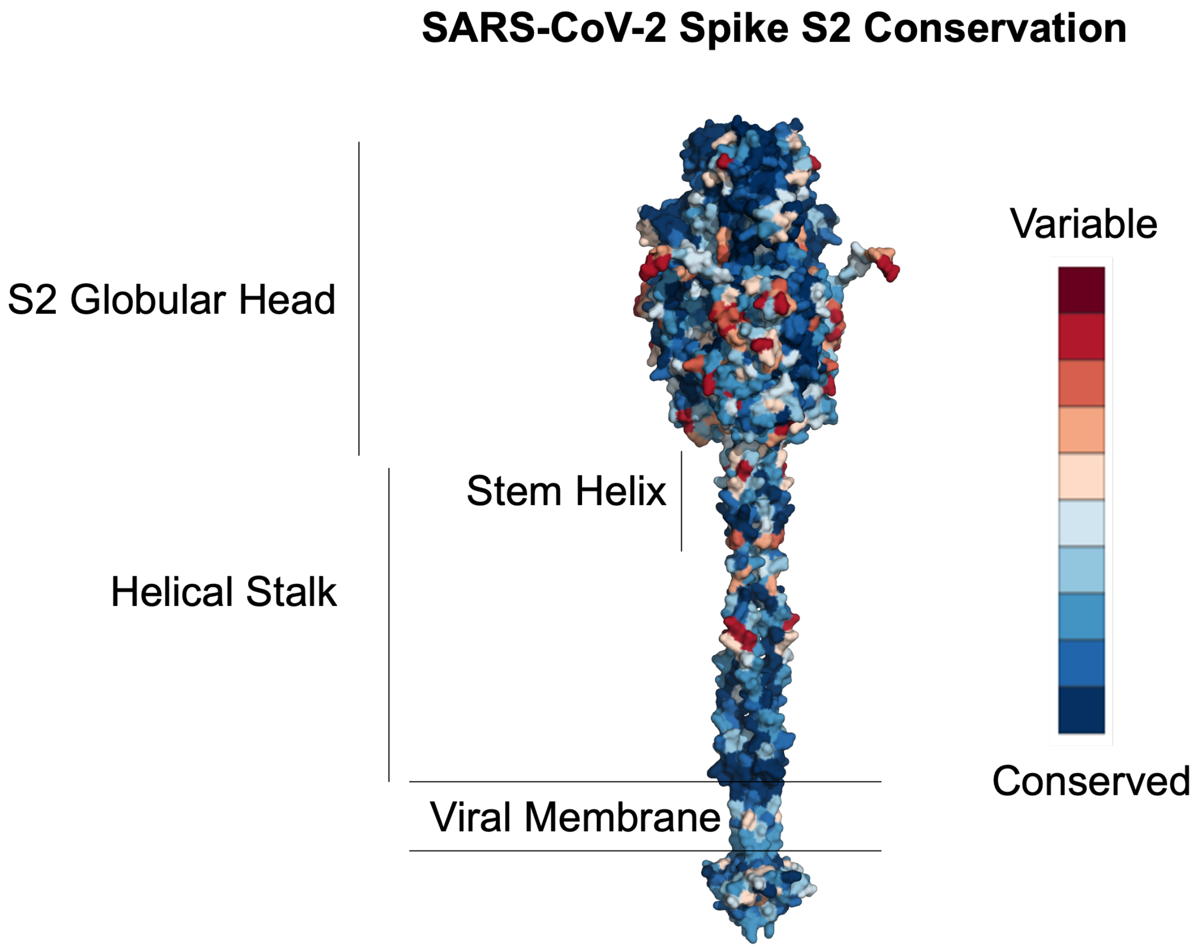

Supplementary Fig. 6. Sequence conservation of the SARS-CoV-2 spike S2 subunit. Sequence conservation of the SARS-CoV-2 spike S2 subunit. Conservation scores generated by the ConSurf[42] webserver. Blue is conserved, red is variable. The alignment file used to produce this image is included as a Supplementary Information file.

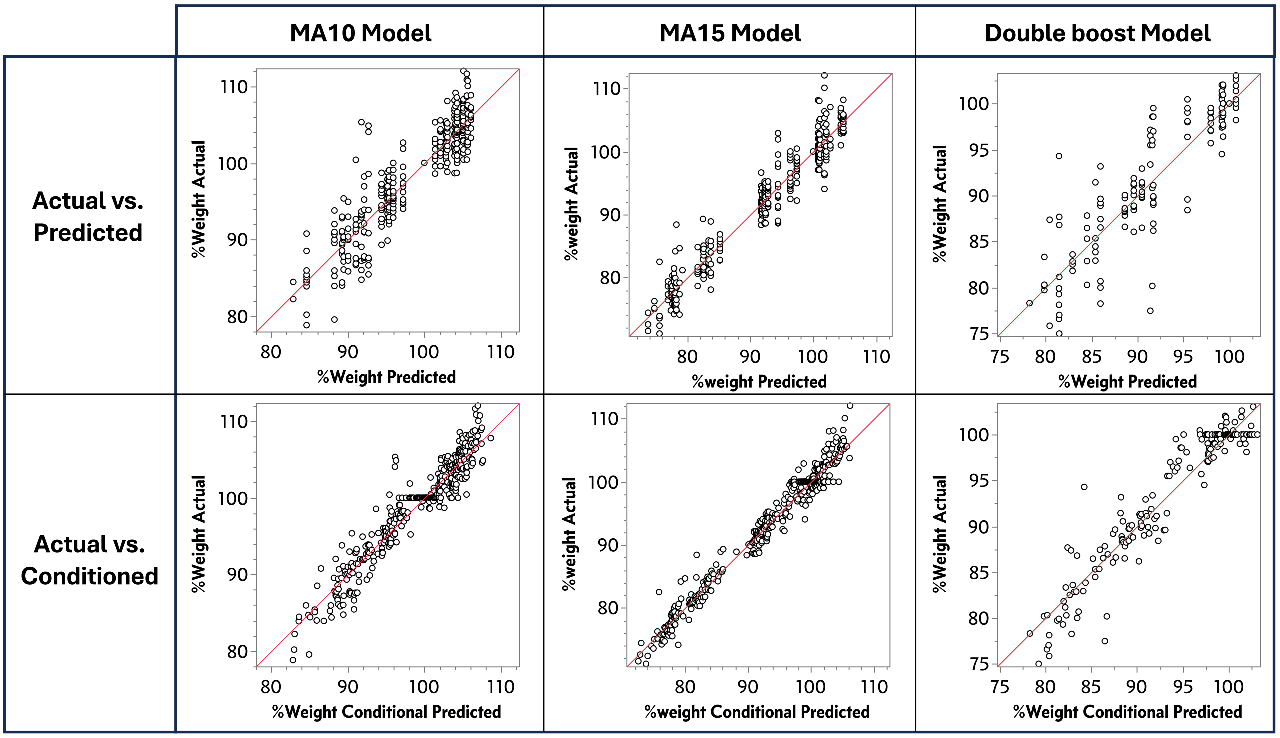

Supplementary Fig. 7. Mixed model performance. We determined the effect of immunogenicity and time on the change in weight for each preclinical experiment using mixed models. Each unique animal was used as a random effect to account for repeated measures. For each mixed model, we visually assessed the model before (actual vs. predicted) and after accounting for variation due to the random effects (actual vs. conditioned) by plotting the observed values of Y against the predicted or conditional predicted values of Y, respectively.

Supplementary Table 1. Characterization of HexaPro S2 variants.

Substitutions	Trimer formation on non-reducing gel	Tm by DSF
Y707C, P792C	yes	triple peaks
Y707C, T883C	yes*	increase 7.7°C
S704C, K790C	yes	increase 7.1°C
A713C, L894C	yes*	increase 13.7°C
Q755C, N969C	no expression	n.d.
G757C, S968C	yes	increase 8.8°C
G891C, P1069C	no expression	n.d.
S1030C, D1041C	no expression	n.d.
G1035C, V1040C	no expression	n.d.

* form mixtures of monomer, dimer and trimer on non-reducing SDS PAGE

n.d. = not determined

Supplementary Table 2. Crystallographic data collection and refinement statistics.
*Values in parentheses are for highest-resolution shell.

	Stabilized S2
**Data collection**	
Space group	*R*3:H
Cell dimensions	
a, b, c (Å)	78.8, 78.8, 478.4
α, β, γ (°)	90, 90, 120
Resolution (Å)	79.92–3.20 (3.42–3.20)*
R_{merge}	0.35 (0.97)
$I / \sigma I$	3.2 (1.8)
$CC_{1/2}$	0.85 (0.52)
Completeness (%)	99.7 (99.7)
Redundancy	4.4 (4.4)
Total reflections	81,107 (14,863)
Unique reflections	18,384 (3,362)
**Refinement**	
Resolution (Å)	67.72–3.20 (3.37– 3.20)
Unique reflections	18,376 (2,634)
Twin operator	k, h, -l
Twin fraction	0.41
R_{work} / R_{free} (%)	22.62/26.19
No. atoms	6,244
Protein	6,230
Water	0
Carbohydrate	61.4
B-factors (Å^2)	
Protein	55.9
Carbohydrate	68.8
R.m.s. deviations	
Bond lengths (Å)	0.002
Bond angles (°)	0.56
Ramachandran (%)	
Favored	95.7
Allowed	4.2
Outliers	0.1

Supplementary Table 3. Cryo-EM data collection and processing.

**EM data collection**			
Microscope		FEI Titan Krios	
Voltage (kV)		300	
Detector		Gatan K3	
Magnification (nominal)		29,000	
Pixel size (Å/pix)		0.81	
Flux (e^-/pix/sec)		10.6	
Frames per exposure		100	
Exposure (e^-/Å^2)		80.5	
Defocus range (μm)		1.5-2.5	
Micrographs collected		4,527	
Micrographs used		3,350	
Particles extracted (total)		1,057,338	
Automation software		SerialEM	
Sample		HexaPro-SS-Δstalk	
**3D reconstruction statistics**			
	Apex closed	Apex partial open	Apex open
Particles	137,403	329,817	277,029
Symmetry	C1	C1	C1
Map sharpening B-factor	-246.4	-190.4	-190.6
Unmasked resolution at 0.143 FSC (Å)	7.5	5.4	6.1
Masked resolution at 0.143 FSC (Å)	4.7	4.2	4.2
