# Supplementary material for: Prefusion-stabilized SARS-CoV-2 S2-only antigen provides protection against SARS-CoV-2 challenge
Source: Nat Commun. 2024 Feb 20;15:1553. doi: 10.1038/s41467-024-45404-x (PMC10879192; doi:10.1038/s41467-024-45404-x)
Supplement: Supplementary file 3 — Reporting Summary [file 41467_2024_45404_MOESM3_ESM.pdf]

nature portfolio

Corresponding author(s): Jason S. McLellan

Last updated by author(s): Jan 17, 2024

Reporting Summary

Nature Portfolio wishes to improve the reproducibility of the work that we publish. This form provides structure for consistency and transparency in reporting. For further information on Nature Portfolio policies, see our Editorial Policies and the Editorial Policy Checklist.

Statistics

For all statistical analyses, confirm that the following items are present in the figure legend, table legend, main text, or Methods section.

n/a	Confirmed	
☐	☒	The exact sample size (*n*) for each experimental group/condition, given as a discrete number and unit of measurement
☐	☒	A statement on whether measurements were taken from distinct samples or whether the same sample was measured repeatedly
☐	☒	The statistical test(s) used AND whether they are one- or two-sided<br>*Only common tests should be described solely by name; describe more complex techniques in the Methods section.*
☒	☐	A description of all covariates tested
☐	☒	A description of any assumptions or corrections, such as tests of normality and adjustment for multiple comparisons
☐	☒	A full description of the statistical parameters including central tendency (e.g. means) or other basic estimates (e.g. regression coefficient) AND variation (e.g. standard deviation) or associated estimates of uncertainty (e.g. confidence intervals)
☐	☒	For null hypothesis testing, the test statistic (e.g. *F*, *t*, *r*) with confidence intervals, effect sizes, degrees of freedom and *P* value noted<br>*Give P values as exact values whenever suitable.*
☒	☐	For Bayesian analysis, information on the choice of priors and Markov chain Monte Carlo settings
☒	☐	For hierarchical and complex designs, identification of the appropriate level for tests and full reporting of outcomes
☒	☐	Estimates of effect sizes (e.g. Cohen's *d*, Pearson's *r*), indicating how they were calculated

Our web collection on statistics for biologists contains articles on many of the points above.

Software and code

Policy information about availability of computer code

Data collection	Serial EM 3.9.0 beta
Data analysis	CCP4i 8.0, iMOSFLM 7.4.0, AIMLESS 0.7.9, PHASER 2.8.3, PHENIX 1.20.1-4487, Coot 0.9.8, GraphPad Prism Version 9.5.1 (528), cryoSPARC v4.3, ChimeraX 1.6.1, Isolde 1.6.0, Data Analysis 11.1

For manuscripts utilizing custom algorithms or software that are central to the research but not yet described in published literature, software must be made available to editors and reviewers. We strongly encourage code deposition in a community repository (e.g. GitHub). See the Nature Portfolio guidelines for submitting code & software for further information.

Data

Policy information about availability of data

All manuscripts must include a data availability statement. This statement should provide the following information, where applicable:

- Accession codes, unique identifiers, or web links for publicly available datasets
- A description of any restrictions on data availability
- For clinical datasets or third party data, please ensure that the statement adheres to our policy

Atomic coordinates and structure factors for the HexaPro-SS-Δstalk crystal structure generated in this study have been deposited in the Protein Data Bank (PDB) under accession code 8U1G. A reporting summary for this article is available as a Supplementary Information file. Source data are provided with this paper.

Research involving human participants, their data, or biological material

Policy information about studies with human participants or human data. See also policy information about sex, gender (identity/presentation), and sexual orientation and race, ethnicity and racism.

Reporting on sex and gender	N/A
Reporting on race, ethnicity, or other socially relevant groupings	N/A
Population characteristics	N/A
Recruitment	N/A
Ethics oversight	N/A

Note that full information on the approval of the study protocol must also be provided in the manuscript.

Field-specific reporting

Please select the one below that is the best fit for your research. If you are not sure, read the appropriate sections before making your selection.

[x] Life sciences [ ] Behavioural & social sciences [ ] Ecological, evolutionary & environmental sciences

For a reference copy of the document with all sections, see nature.com/documents/nr-reporting-summary-flat.pdf

Life sciences study design

All studies must disclose on these points even when the disclosure is negative.

Sample size	Sample sizes were chosen to minimize the number of mice used in the study while providing sufficient statistical power to observe significant differences.
Data exclusions	No data were excluded.
Replication	We did not attempt to replicate the mouse immunization studies or the initial characterization of protein expression for single-substitution variants. Ten to fifteen biological replicates in a single immunization experiment is sufficient to observe significant differences. To reduce the number of mice being sacrificed in this study, repeating the animal experiment is not necessary. For the protein characterization, inclusion of the base construct in each expression set allowed for enough information to determine which constructs to move forward with.
Randomization	Mice were randomly assigned to experimental groups. For the protein characterization, variants were grouped based on design strategy for direct SEC comparison. Each variant plasmid and the base construct were randomly assigned to the transfection groups.
Blinding	Investigators were blinded to group allocation during data collection

Reporting for specific materials, systems and methods

We require information from authors about some types of materials, experimental systems and methods used in many studies. Here, indicate whether each material, system or method listed is relevant to your study. If you are not sure if a list item applies to your research, read the appropriate section before selecting a response.

Materials & experimental systems

n/a	Involved in the study	
[x]	[ ]	Antibodies
[ ]	[x]	Eukaryotic cell lines
[x]	[ ]	Palaeontology and archaeology
[ ]	[x]	Animals and other organisms
[x]	[ ]	Clinical data
[x]	[ ]	Dual use research of concern
[x]	[ ]	Plants

Methods

n/a	Involved in the study	
[x]	[ ]	ChIP-seq
[x]	[ ]	Flow cytometry
[x]	[ ]	MRI-based neuroimaging

Eukaryotic cell lines

Policy information about cell lines and Sex and Gender in Research

Cell line source(s)	FreeStyle 293-F cells (ThermoFisher, Cat #R79007) Vero cells (ATCC, CCL-81)
Authentication	FreeStyle-293F cells were purchased commercially and were not further validated. Vero cells were not authenticated.
Mycoplasma contamination	Confirmed that all cell lines are free of mycoplasma by PCR
Commonly misidentified lines (See ICLAC register)	No commonly misidentified cell lines were used in the study.

Animals and other research organisms

Policy information about studies involving animals; ARRIVE guidelines recommended for reporting animal research, and Sex and Gender in Research

Laboratory animals	BALB/cAnNHsd (BALB/c) mice (10 weeks old). Mice were kept at a 12h light / dark cycle (7am-7pm), temperature was between 20-23 C, humidity was between 30-70% (usually in the 50s)
Wild animals	No wild animals were used in the study.
Reporting on sex	In this study female BALB/c mice were used because most historical data was obtained from female mice and experiments comparing female and male mice did not show significant sex-dependent differences in our hands within this age distribution. Furthermore, female mice were used because of logistical and biosafety considerations like reduced fighting among cage mates and reduced risk for laboratory incidents in the BSL3. Including male mice would have doubled the study size which we tried to avoid due to animal welfare reasons and biosafety considerations at the BSL3. More specifically, we avoided male mice due to increased aggression levels in males and thereby reducing the risk of animal bites and laboratory acquired infections by workers.
Field-collected samples	No field collected samples were used in the study.
Ethics oversight	All in vivo experiments described were approved by the Institutional Animal Care and Use Committee at the University of North Carolina at Chapel Hill (animal protocol number: 21-056) in agreement with guidelines outlined by the Association for the Assessment and Accreditation of Laboratory Animal Care and the U.S. Department of Agriculture

Note that full information on the approval of the study protocol must also be provided in the manuscript.
